# Deforestation, drainage network, indigenous status, and geographical differences of malaria in the State of Amazonas

**DOI:** 10.1186/s12936-015-0859-0

**Published:** 2015-09-30

**Authors:** Wagner Cosme Morhy Terrazas, Vanderson de Souza Sampaio, Daniel Barros de Castro, Rosemary Costa Pinto, Bernardino Cláudio de Albuquerque, Megumi Sadahiro, Ricardo Augusto dos Passos, José Ueleres Braga

**Affiliations:** Fundação de Vigilância em Saúde do Amazonas, Manaus, Brazil; Escola Nacional de Saúde Pública Sergio Arouca, FIOCRUZ, Rio de Janeiro, Brazil; Instituto de Medicina Social, Universidade do Estado do Rio de Janeiro, Rio de Janeiro, Brazil; PVS PECTI-SAÚDE/Fundação de Amparo à Pesquisa do Estado do Amazonas (FAPEAM), Manaus, Amazonas Brazil

**Keywords:** Malaria, Deforestation, Drainage network, Indigenous status, Brazilian Amazon

## Abstract

**Background:**

Malaria is a major public health problem worldwide. In Brazil, an average of 420,000 cases of malaria have been reported annually in the last 12 years, of which 99.7 % occurred in the Amazon region. This study aimed to analyse the distribution of malaria in the State of Amazonas and the influence of indigenous malaria in this scenario, to evaluate the correlation between incidence rates and socio-economic and environmental factors, and to evaluate the performance of health surveillance services.

**Methods:**

This ecological study used secondary data obtained from the SIVEP-MALARIA malaria surveillance programme. The relationship between demographic, socio-economic and environmental factors, the performance of health surveillance services and the incidence of malaria in Amazonas, a multiple linear regression model was used.

**Results:**

The crude rate of malaria in Amazonas was 4142.72 cases per 100,000 inhabitants between 2003 and 2012. The incidence rates for the indigenous and non-indigenous populations were 12,976.02 and 3749.82, respectively, with an indigenous population attributable fraction of only 8 %. The results of the linear regression analysis indicated a negative correlation between the two socio-economic indicators (municipal human development index (MHDI) and poverty rate) and the incidence of malaria in the period. With regard to the environmental indicators (average annual deforestation rate and percentage of areas under the influence of watercourses), the correlation with the incidence rate was positive.

**Conclusions:**

The findings underscore the importance of implementing economic and social development policies articulated with strategic actions of environmental protection and health care for the population.

## Background

Malaria is a major public health problem worldwide, with approximately 198 million cases and 584,000 deaths in 2013. This endemic disease occurs predominantly in Asia, Africa, and Latin America [1]. In Brazil, an average of 420,000 cases of malaria have been reported annually in the last 12 years, of which 99.7 % occurred in the Amazon region [2].

Malaria is influenced by socio-economic and environmental factors, including forest cover, drainage network, rainfall, poverty level, economic inequality, and level of education of the population [3–6]. Recent studies using computer modelling have focused on the interaction of climate and hydrological aspects with the life cycle of the vector and host to explain the dynamics of malaria transmission [7]. To date, there is no consensus in the literature regarding the effects of deforestation on malaria transmission. However, some authors have identified a correlation between deforestation and high malaria incidence rates [8–15], whereas other authors have reported an increased number of malaria cases in the forest fringes [16–19].

In addition, the presence of indigenous people has been identified as an explanatory factor for the maintenance of endemicity in some areas [20, 21], particularly in the Amazon region [22, 23].

In 2010, the Brazilian indigenous population consisted of 817,963 individuals. These individuals can be found across the country but are more concentrated in the northern region, where 37 % of Brazilian Indians are located. The State of Amazonas has 168,680 Indians, accounting for 5 % of the general population of the State and for more than 20 % of the indigenous population of Brazil [24].

According to Santos and Coimbra Jr, Brazilian Indians exhibit social and cultural characteristics that make them vulnerable to various diseases and disorders, including malaria [25]. As a consequence of this vulnerability, approximately 16,000 cases of malaria among Brazilian Indians have been reported to the Amazonas Health Surveillance Foundation (FVS) in the past ten years [26]. However, the effect of indigenous malaria on disease transmission in Amazonas remains unknown.

The present study aimed to increase knowledge of the social and environmental determinants of malaria in the Amazon region and to enable the development of more specific and effective strategies for disease control. Moreover, it aimed to understand the dynamics of malaria in the indigenous population in Amazonas and how that relates to malaria in the general population, while controlling for other environmental and socioeconomic factors and the performance of health services.

## Methods

This ecological study used secondary data from the epidemiological surveillance system for malaria; the municipalities and regional health administrations in the State of Amazonas were used as the units of analysis.

**Fig. 1:**
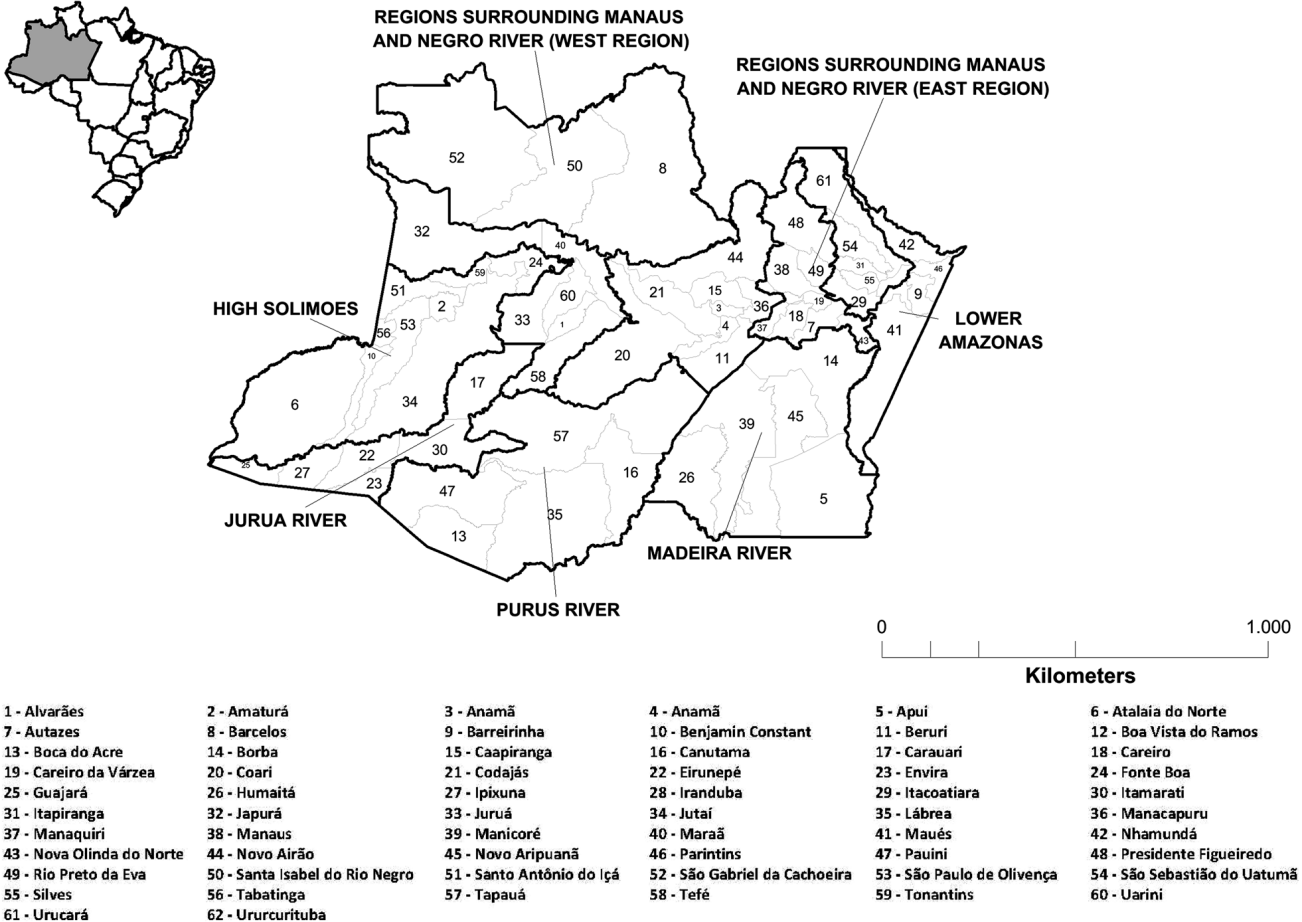
Municipalities and regional health administrations in the State of Amazonas

Amazonas is located in the northern region of Brazil; it has a total area of 1,559,161 sq km and comprises 62 municipalities distributed in nine regional health administrations. The State has 3,483,985 inhabitants, of whom 1,802,525 live in the capital, Manaus [27]. For this study, the area surrounding Manaus and the Negro River was subdivided into east and west regions. The east region comprises the cities of São Gabriel da Cachoeira, Santa Isabel do Rio Negro and Barcelos, whereas the west comprises the remaining nine municipalities of this region (Fig. 1).

The number of malaria cases was obtained from the Information System for the Epidemiological Surveillance of Malaria (MALARIA-SIVEP) and was made available by the FVS of the Department of Health of Amazonas.

It was considered for this study, the Ministry of Health malaria case definition (malaria suspect person whose presence of parasite or some of its components have been identified in the blood for laboratory examination) [28]. Only new malaria cases (i.e. excluding relapsing cases and treatment failure) reported between January 1, 2003 and December 31, 2012 in the population living in Amazonas were selected. Both active and passive surveillance cases were taken into account. Data about the parasite specie was not considered, since the proportion of falciparum cases was thought to be irrelevant in the current epidemiologic scenario [29]. Malaria cases were classified according to ethnicity: indigenous or non-indigenous. The cases were considered indigenous when the records were obtained from locations designated “village/maloca”. Regardless being a limitation, this strategy has been used in other research [30]. The cases classified as non-indigenous were obtained from the following locations: “neighbourhood”, “farm”, “town”, “rubber plantation”, “sawmill”, and other categories present in the location database.

To calculate the average annual incidence rates, all new cases of malaria reported during the study period were added, divided by the sum of the estimated population for each year, and multiplied by 100,000.

Because the study aimed to evaluate the influence of indigenous malaria on global malaria (which occurs in the general population) in Amazonas, the incidence rates among indigenous and non-indigenous populations were calculated. Subsequently, the proportion of outcomes (malaria cases) assigned to the indigenous groups was calculated using the population attributable fraction (PAF), which is a measure widely applied in public health. This measure is often adopted to determine the effect of the elimination of the risk factor for a certain outcome, thus allowing the measurement of how much the incidence of malaria in Amazonas can be reduced if all malaria cases in the indigenous population were eliminated.

The PAF for malaria associated with the indigenous groups was calculated as a function of the relative risk according to the following formula:

$$PAF = \frac{{n_{11} }}{{n_{0.1} }} \times \frac{RR - 1}{RR}$$

In which RR is the relative risk of becoming ill from malaria among indigenous people compared with that among non-indigenous people, n_11_ is the number of indigenous people who became ill from malaria, and n_0.1_ represents the total number of individuals who became ill from malaria.

Six sociodemographic indicators were evaluated: (1) the municipal human development index (MHDI), which is the geometric mean of the indices of the dimensions income, level of education and longevity, with equal weights, in addition to the individual dimensions; (2) the Gini index for income, which measures the degree of inequality in the distribution of individuals according to per capita household income; (3) the poverty rate, which expresses the percentage of individuals with a per capita household income less than half the minimum wage in that period; (4) the income ratio, which compares the average per capita income of individuals belonging to the richest tenth of this distribution with the average per capita income of individuals belonging to the poorest two-fifths; (5) the average household income per capita, which is the ratio between the sum of the income of all individuals living in permanent private households and the total number of individuals; and, (6) the unemployment rate of the population aged ≥10 years, which is the percentage of the economically active population (EAP) in this age group that was unemployed. These indicators were obtained from the United Nations Development Programme.

To evaluate the malaria surveillance capacity in the municipalities, two types of indicators were selected: an indirect measure of the performance of health surveillance services, designated as the SUS Performance Index, which is composed of access and effectiveness indicators [31] and direct measures of the performance of health surveillance services recommended by the Ministry of Health [32]. In the latter group, the following variables were selected: (a) percentage of cases of falciparum malaria; (b) percentage of cases in which the period between the first symptoms and the administration of treatment was less than 48 h; (c) percentage of individuals protected by long-lasting, insecticide-treated mosquito nets; and, (d) average annual number of laboratories that were established and active in the municipalities. These indicators were calculated using MALARIA-SIVEP data.

The three environmental indicators studied were (a) average annual deforestation rate between 2003 and 2011; (b) average annual forest cover in the municipalities between 2003 and 2011, which was measured by the average annual percentage of the municipal area that harbours forest vegetation; and, (c) percentage of areas under the influence of watercourses in 2010. Data on deforestation and forest cover were obtained from the Satellite Surveillance Programme of the Brazilian Amazon Rainforest and the National Institute for Space Research. This data was obtained from the imaging of 30 meters resolution TM/LANDSAT images using the SPRING computational environment. The image selection taken into account cloud cover and dates closest to August 1st of each year in order to compose the scene from which the deforestation data were generated. To calculate the percentage of areas under the influence of watercourses, a buffer area of a 150-m radius was considered around the drainage network of the municipality using ArcMap software version 10.1 [33]. This indicator is obtained by dividing the areas of influence of the drainage network by the total area of the municipality. The digital images of the drainage network were obtained from the Hydrological Information System of the National Water Agency [34], and the digital cartographic images were obtained from the Brazilian Institute of Geography and Statistics (IBGE).

Data on the populations living in municipalities in Amazonas were obtained from the 2000 and 2010 Censuses and the intercensus projections developed by the IBGE. Data on the indigenous population living in the municipalities were obtained from the Information System of Indigenous Health Care of the Ministry of Health.

To study the relationship between demographic, socioeconomic, and environmental factors, the performance of health surveillance services and the incidence of malaria in Amazonas, a multiple linear regression model was used. It was also appreciated collinearity and the interaction between the explanatory variables in the analysis that led to the final multiple regression model. For the final model, only the variables associated with the outcome at a significance level of 0.20 using the *backward stepwise* technique were selected. Therefore, the values of the association between the average annual incidence rate and demographic, socioeconomic, and environmental indicators, as well as the PAF of malaria for the indigenous population, were calculated. The statistical analyses were performed using the STATA statistical package version 13, and ArcGIS software version 10.1 was used for the creation of the thematic maps.

## Results

Between 2003 and 2012, the crude rate of malaria in Amazonas was 4142 cases per 100,000 inhabitants. Although the incidence rate was high in all regions, the incidence was not homogeneous throughout the State. The average annual rate in the region surrounding Manaus and Rio Negro (east) was 12,407 cases per 100,000 inhabitants, compared to 358 cases per 100,000 inhabitants in the lower Amazon region (Table 1).

**Table 1:** Incidence rates of malaria among the indigenous and the non-indigenous population, and the population attributable fraction of malaria among the indigenous population in Amazonas and surrounding regions

State/region	Total population	Population of indigenous people	Population of non-indigenous people	Overall annual average incidence rate	Annual average incidence rate among the indigenous population	Annual average incidence rate among the non-indigenous population	Indigenous population attributable fraction (%)
Amazonas	3341,143	126,236	3214,907	4142.72	12,976.02	3749.82	8.0
Alto Solimoes	230,744	47,496	183,248	4497.66	13,210.45	2239.37	50.0
Lower Amazonas	212,716	5418	207,298	358.13	996.59	341.44	5.0
Regions surrounding Manaus and Negro River (east)	1928,278	10,761	1917,516	2978.17	6819.55	2956.61	1.0
Regions surrounding Manaus and Negro River (west)	78,565	38,929	39,636	12,407.85	14,051.52	10,793.47	13.0
Middle Amazonas	144,074	368	143,706	3760.7	39,647.22	2638.65	3.0
Jurua River	113,439	3569	109,870	7255.33	18,262.31	6897.78	5.0
Madeira River	152,488	8274	144,214	8223.04	9911.53	8126.17	1.0
Negro River and Solimoes	241,341	2676	238,665	5800.41	12,268.38	5727.87	1.0
Purus River	109,802	4892	104,910	9644.27	23,802.81	8983.95	7.0
Triangulo	129,697	3853	125,844	7188.02	19,138.33	6822.13	5.0

The incidence rate among the indigenous population was 12,976 cases per 100,000 inhabitants, compared to 3749 cases per 100,000 inhabitants among the non-indigenous population. In all regions of the State, incidence rates were higher among the indigenous population compared to the non-indigenous population. Of note, in middle Amazonas, the incidence rate among the indigenous population was 15 times higher than that among the non-indigenous population.

Although the PAF of malaria for the indigenous population was only 8 % in the State, this indicator was very high in some regional health administrations. In the Alto Solimões region, approximately 50 % of the malaria cases were attributed to malaria in the indigenous population. In other regions, including the areas surrounding Manaus and the Negro River (east), the Madeira River and the Negro River and Solimões, the PAF was only 1 % (Table 1).

Factors associated with the performance of health surveillance services and other sociodemographic and environmental factors were not included in the regression model because they showed no correlation with the outcome at a significance level of 0.20.

**Fig. 2:**
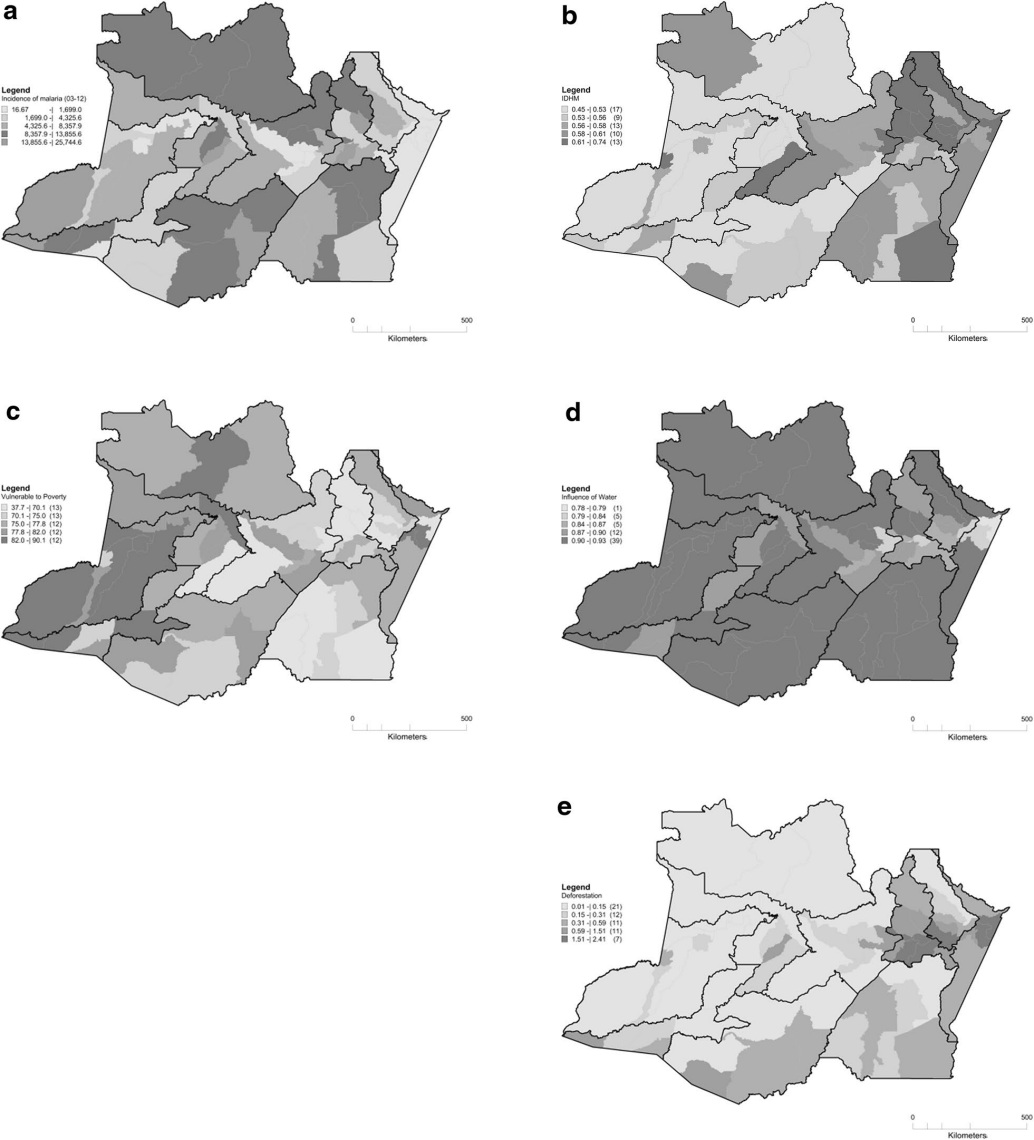
Spatial distribution of the malaria incidence and risk factors in the State of Amazonas. **a** Incidence rate of malaria between 2003 and 2012; **b** municipal human development index in 2010; **c** proportion of people vulnerable to poverty in 2010; **d** proportion of areas under the influence of watercourses; and **e** average annual deforestation rate between 2003 and 2012

In the municipalities of Amazonas, the MHDI varied between 0.45 in Atalaia do Norte and 0.74 in Manaus. The largest MHDI values occurred in the east of the State in Manaus and adjacent municipalities, whereas the lowest values were observed in the municipalities surrounding Manaus and the Negro River (west), Alto Solimões, and the Purus River (Fig. 2b).

The poverty rate varied between 38 % in Manaus and 90 % in Itamarati. With the exception of the municipalities surrounding Manaus, which were strongly influenced by the conditions in the capital, and a few other municipalities, including Coari and Tefé, the remaining municipalities had a medium or high poverty rate (Fig. 2c).

In Amazonas, which has an immense drainage network, a high proportion of areas were under the influence of watercourses. Despite the high density of watercourses throughout the State, only a few municipalities surrounding Manaus, the Negro River and lower Amazonas had low percentages of areas under the influence of watercourses compared to the other municipalities (Fig. 2d).

With regard to the average annual deforestation rate between 2003 and 2012, the municipalities in the eastern and southern regions of the State had the highest deforestation rate. In the remaining municipalities, the rate was relatively low except for Tabatinga and Guajará, which are located in the western and south-western regions of the State, respectively, where the rates were higher than those in the neighbouring municipalities (Fig. 2e).

The multiple linear regression analysis indicated a negative correlation between the incidence rate and two social indicators: MHDI and the poverty rate. In addition, the model used indicated a positive correlation between the incidence rate and two environmental indicators: the average annual deforestation rate and the percentage of areas under the influence of watercourses. The other environment and sociodemographic and health services performance indicators were not related to the outcome (malaria incidence rate) in the final multiple regression model. Considering the value of the coefficient of determination (R^2^), this model explained 35 % of the variation in the incidence rate observed in the period (Table 2).

**Table 2:** Factors associated with malaria in the municipalities of Amazonas between 2003 and 2012

Factor	Regression coefficient	*p* value	95 % CI
MHDI	−81,635.66	0.000	−125,164.70; −38,106.64
Poverty rate	−317.47	0.012	−561.82; −73.13
Average annual deforestation rate	4205.19	0.003	1490.98; 6919.40
Percentage of areas under the influence of watercourses	75,288.28	0.001	31,701.63; 118,874.90

## Discussion

Malaria occurs throughout Amazonas, but its distribution in the State is heterogeneous. The results indicated that the risk of acquiring malaria among the indigenous population was three times greater than that among the non-indigenous population, corroborating previous studies that revealed the susceptibility of this group to the endemic disease [35, 36]. Nevertheless, the incidence of malaria in the State was little influenced by malaria among the indigenous population. Moreover, the findings indicated that the pattern of distribution of the disease was determined by the MHDI, the poverty rate, the drainage network, and the average annual deforestation rate, confirming the strong relationships of this endemic disease with socioeconomic and environmental factors [3, 8, 10, 37, 38].

For a long time, the isolation of riverine communities favoured the control of malaria by limiting the spread of the disease. In recent years, with the development of social inclusion policies and microfinance projects, such as the government initiative to distribute engines for boats, a significant increase in migration has been observed in the State [39]. Previous studies have shown that migration can encourage the introduction or re-introduction of malaria and thereby hinder its control [35, 36, 40]. According to these authors, the high incidences of malaria in the Amazon state may be partly justified by these factors. It is likely that the large distances between the municipality headquarters and riverine communities, combined with the limited mobility in the region, present challenges for the implementation of a health service network that adequately and effectively serves the entire population.

With regard to indigenous peoples, it is known that poor sanitation and housing, in addition to low coverage and quality of health care services, are responsible for the worsening and deteriorating health of Brazilian Indians [41]. The results of this study corroborate these findings by indicating that the indigenous population had a higher risk of malaria compared to the non-indigenous population in all regions of the State. In the middle Amazonas region, the malaria incidence rate among the indigenous population was 15 times greater than that among the non-indigenous population.

Although Amazonas has a large number of indigenous people who are vulnerable to malaria, the results indicated that the incidence rate of malaria in the State was little influenced by indigenous malaria. It found that indigenous malaria represented only 8 % of the total cases of malaria in the State. However, in Alto Solimões and areas surrounding Manaus and the Negro River (west), the PAFs of malaria in the indigenous population were 50 and 13 %, respectively. These findings reinforce the importance of developing policies and strategies aimed at the prevention and control of malaria among the indigenous population in these regions.

A negative correlation was observed between the incidence rate of malaria and the MHDI, but that does not necessarily imply causality because the population with a lower MHDI may be more susceptible to the disease, or the disease itself may hinder the socio-economic development of the community [38]. The model adopted also indicated a negative correlation between the incidence rate and the poverty rate. In this respect, the percentage of the population living in poverty in Amazonas is very high. In most municipalities in the State, the poverty rate is greater than 25 %, except in Manaus, where the rate is 13 %. Possibly, in the municipalities with the lowest poverty rates, the population may have greater access to diagnostic services, and consequently, more cases of malaria were reported. Considering that poverty was associated with the level of education, another explanation is that in conditions of lower poverty and higher levels of education, the population more often seeks diagnostic services, therefore, malaria cases are more commonly reported.

No correlation was observed between the incidence rate of malaria and the performance of health surveillance services, most likely because these indicators are associated with the performance of the municipal primary health care system, which is poorly integrated with the actions of diagnosis and treatment of malaria [42]. In addition, more specific indicators of the performance of malaria control actions were not associated with the incidence rate of the disease. Furthermore, the percentage of cases reported for which the period between the first symptoms and the administration of treatment was less than 48 h may not indicate that treatments are performed in a timely manner because this condition was self-reported and of low reliability. Similarly, the average annual number of established and active laboratories does not consider the size of the target population of each area, which may explain the lack of correlation. Furthermore, the proportion of individuals protected by mosquito nets could increase in areas of higher incidence, indicating the need for the adoption of control measures, or could indicate the effect of this intervention, leading to the reporting of fewer cases. The failure to identify the correlation between the percentage of cases of falciparum malaria and the incidence rate could be explained by the context of this study, in which more than 10 % of the cases were a result of *Plasmodium falciparum* in 92 % of the municipalities.

The results support the existence of a correlation between the presence of watercourses and the malaria transmission cycle. To detect such a correlation, it used an indicator based on the area of influence of the drainage network of each municipality. It is of note that the analysis did not consider the characteristics of the watercourses with regard to their potential for vector development and their proximity to inhabited areas.

It was also found a correlation between the incidence rate of malaria and the average annual deforestation rate, corroborating the results of other studies [8, 37]. Although the deforestation rate in Amazonas is one of the lowest compared to other states in the Amazon region, both the number of ecologically favourable breeding sites for vector proliferation [9, 10] and the number of malaria cases [6, 11] increase on the forest fringes under deforestation.

The limitations of this study include the possibility of underreporting malaria cases, either because of problems related to coverage and access to services offered to the population and possible errors in classification and/or diagnosis of malaria cases reported in Amazonas. Despite these limitations, the findings are useful for making a public health decision, as they indicate areas of priority for the development of actions aimed at the prevention and control of malaria.

## Conclusions

This study reinforces the importance of socio-economic and environmental factors in the aetiology of malaria in the State of Amazonas. Therefore, economic and social policies should be implemented in conjunction with strategic actions for environmental protection and health surveillance of the population.
